# Practical nursing students' learning and assessment during work‐based placement: A qualitative study

**DOI:** 10.1002/nop2.1849

**Published:** 2023-05-23

**Authors:** Välimaa Virpi, Tuomikoski Anna‐Maria, Mikkonen Kristina

**Affiliations:** ^1^ Research Unit of Health Sciences and Technology University of Oulu, Oulu; Vaasa University of Applied Sciences Vaasa Finland; ^2^ Oulu University Hospital, The Wellbeing Services County of North Ostrobotnia, Oulu; Research Unit of Health Sciences and Technology University of Oulu Oulu Finland; ^3^ Research Unit of Health Sciences and Technology University of Oulu, Oulu, Medical Research Center Oulu, Oulu University Hospital and University of Oulu Oulu Finland

**Keywords:** assessment, learning, mentor, practical nurse, student, vocational education, work‐based placement

## Abstract

**Aim:**

To describe practical nursing students', mentors' and educators' perceptions of student learning and assessment of learning progress during work‐based learning.

**Design:**

A qualitative descriptive study.

**Methods:**

The research data were collected by interviewing eight practical nursing students, 12 mentors and eight educators (total *n* = 28) from three vocational institutions and four social‐ and health care organizations in Finland during November 2019–September 2020. The interviews were conducted as focus group interviews, after which the collected material was subjected to content analysis. The researchers had received appropriate research permits from the target organizations.

**Results:**

Work‐based learning depends on the student, who must be goal‐oriented and responsible for their own learning. The mentor also plays a key role in the learning process as the supporter and enabler of a student's goal‐oriented learning process. The educator is responsible for instructing both students and mentors, and supporting a student's goal‐oriented learning process. The vocational institution also has a role in successful learning among practical nursing students as an enabler of students' individual learning process. The participants shared that the workplace is responsible for ensuring a secure learning environment.

## 1 INTRODUCTION

Commonly, practical nursing studies in vocational institution includes work‐based learning in addition to theoretical studies. The concept of work‐based learning or on‐the‐job learning is used when the practical learning environment is discussed in the context of secondary vocational education (MEC, 2019). Vizcaya‐Moreno et al. (2018) have previously stated that clinical learning, which is seen as work‐based learning in this study, significantly impacts the formation of students' knowledge, skills and attitudes. As such, practical work environments which include high‐quality student mentoring and assessment will result in motivated and skilled graduating health professionals (Helminen et al., 2016; Pitkänen et al., 2018; Tuomikoski et al., 2020a).

There are only a few countries where practical nurses complete their studies at vocational institutions (MEC, Ministry of Education and Culture, Finnish National Agency for Education, 2019). For instance, in Finland, a practical nurse is a regulated profession under both the Act and Degree on Health Care Professionals (FNAE, 2017) and the Act on Social Care Professionals (FNAE, Finnish National Agency for Education., 2017). Practical nurse education and training cover 180 competence points. The duration of the education is 3.5 years, but the student's previous competence may affect the duration by shortening it individually. (FNAE, Finnish National Agency for Education., 2017). A significant portion of the studies is carried out with training or an apprenticeship agreement in real‐work environments, for example, in kindergarten, school, service house, home care or hospital (MEC, Ministry of Education and Culture, Finnish National Agency for Education, 2019). The learning at the vocational institution influences learning at the work‐based placements and vice versa (Goh, 2014). After graduation, regulated practical nurses can also work in the aforementioned workplaces. In England, for example, ‘health care assistants’ and ‘clinical support staff’, who do not have well‐defined training requirements, perform certain work tasks that, in Finland, are given to practical nurses (NHS, 2022). However, the nursing associate (NAS) role has been introduced in England in recent years to bridge the gap between health care assistants (HCAs) and registered nurses (RNS). Nursing associates have gained two Nursing Associate Foundation Degree, typically involving 2 years of higher‐level study (King et al., 2020, 2022). In Sweden, students can study to be a practical nurse in high school or complete similar studies via adult education (Gymnasieguiden., 2021; Komvuxutbildningar, 2021). In Australia these second‐level nurses undergo 2 years of training and are known as ‘Enrolled Nurses’ (Aussizz group, 2023) and in North America they are often called ‘Licensed Practical Nurses’ (Nance & Brown, 2022).

In Finland, a personal competence development plan (PCDP) is drawn up for each student during practical nurse training. This plan includes the student's prior competence, specifies which knowledge and skills the student still needs, and outlines how, and in which learning environment(s), these competences can be developed. During the education, the student acquires competence during work‐based learning through either a training agreement or an apprenticeship. Students have 4–5 periods of work‐based learning depending on their prior competence. During work‐based learning, the mentor, the educator and the entire work community give the student regular feedback on the progress of the student's learning. The learning goals and assessment of learning are based on national vocational requirements and assessment criteria. Following the completion of work‐based learning, the student will demonstrate his/her competence during a competence demonstration. The assessment of a student's competence is decided jointly by the educator and the representative of working life (mentor/assessor).

It is essential that the mentors/assessors and educators are able to distinguish the assessment of learning from the assessment of competence (FNAE, 2018). The mentor/assessor who is nominated for a practical nurse student is usually also a practical nurse; however, other health care professionals who have graduated from a university of applied sciences, e.g., a registered nurse, can act as a mentor. It is important that there are enough proficient mentors in the workplace so that each student can be assigned a mentor for work‐based learning (Pitkänen et al., 2018). The educator in this study means the nurse teacher, who is employed by the vocational institution. The educator works mainly at a vocational institution, but she/he is responsible for the support and instruction of both the student and the mentor in the workplace.

Previous literature has highlighted several aspects of mentoring that influence the success of nursing student learning in the clinical learning environment. For example, the mentor has an important role in student learning and assessment because a large number of health students are present in the practical learning environment (Arkan et al., 2018; Kälkäjä et al., 2016) and the resources for mentoring have decreased significantly (Kälkäjä et al., 2016). According to Papastavrou et al. (2016), students estimate the mentor‐student relationship as the most influential factor in student satisfaction in a clinical learning environment. Studies have also shown that mentoring education impacts their competence and assessment skills; for this reason, it is crucial to regularly organize mentoring education (Immonen et al., 2019; Tuomikoski et al., 2018, 2020b). Collaboration between the student, mentor, and educator during clinical training is important (Dimitriadou et al., 2015; Helminen, 2017; Hyvärinen et al., 2019). In addition to workplace visits, the educator can contact the workplace by e‐mail or phone (Helminen, 2017). Some educational institutions also use an e‐learning environment, which allows the educator to mentor the student in cooperation with the mentor. However, educators' visits to the workplace are still considered a very important factor (Hyvärinen et al., 2019). In addition, mentors value receiving support for student assessment from their organization, superiors and work community (Hyvärinen et al., 2019). This support also exerts positive effects on the pedagogical atmosphere (Pitkänen et al., 2018). A mentor's motivation, professionalism, interpersonal skills and pedagogical knowledge contribute to the successful assessment of students (Kälkäjä et al., 2016; Karjalainen et al., 2015; Tuomikoski et al., 2018). Motivated mentors will hold regular reflective meetings with students during which they can ask and gauge each student's progress. Reflective discussions have been found to positively affect student learning and satisfaction (Pitkänen et al., 2018; Tuomikoski et al., 2018). However, studies on the clinical learning environment and mentoring largely focus on nursing students, with no or little focus given to practical nurses. With health care systems facing a crisis, i.e., global shortages in nurses, practical nurses could play an essential role in ensuring basic patient care. The workplace as a learning environment must be understood as a multi‐factorial concept which involves various roles for the people and organizations participating in this form of teaching (Billett, 2004; Goh, 2021). We have considered the importance of the clinical learning environment and mentoring on students' learning and assessment, and aimed to answer the following research question: what kinds of perceptions do practical nursing students, mentors and educators have of student's work‐based learning and the assessment of learning progress?

## 2 METHODS

### 2.1 Study design

The research applied a qualitative, descriptive research design with inductive content analysis. The qualitative research method was chosen because the assessment of learning and competence of nursing students in the workplace is a little‐studied area (Kyngäs, 2020b).

### 2.2 Participants

The study participants were selected through purposive sampling relating the studied phenomenon represented in the research question (Bowling, 2014), more precisely, with 8 practical nursing students, 12 mentors and 8 educators (total *n* = 28) from 3 vocational institutions and four social‐ and health care organizations in Finland. The number of participants was based on data saturation and information power. The students who participated in the study were in their final year of studies and had completed 2–4 work‐based learning periods. The mentors who participated in the study were practical nurses who had 6–36 years of work experience, with all having mentored students at some point in their careers. They worked in home care, service housing and institutional care. The educators who participated in the study had 6–30 years of work experience, and all of them had mentored students at some point in their work as an educator. The educators worked in a vocational institution that offers upper secondary vocational education in Finland. The participants were recruited for the study by the contact person of each organization included in the study. The contact persons informed by e‐mail all students, mentors and educators about the study. The study included voluntary students who had completed at least one period of work‐based learning. The mentors and educators enrolled in the study had to have experience in mentoring students in work‐based placement.

The number of participants was based on data saturation and information power. Data saturation was reached when themes and categories became repetitive, and the new data yielded redundant information (Polit & Beck, 2021). Malterud et al. (2016) have identified five items, that have an impact on the information power of the sample: (a) study aim, (b) sample specificity, (c) use of established theory, (d) quality of dialogue and (e) analysis strategy. In this study, the collected data achieved the aim of the study. The study was limited to the practical nursing students' learning and assessment of it in the specific context, in the workplace. The study's theoretical background was research literature related to the learning and assessment of nursing students in a clinical learning environment. The participants in the study had experience and knowledge about the research phenomenon and, were able to report their perceptions in a very diverse way. The researcher who interviewed the participants has worked as an educator for practical nurses at the vocational institution for 8 years and has a deep knowledge of the research phenomenon. As a result, the researcher could identify the information power during the data collection and analysis process.

### 2.3 Data collection

The interviews were conducted as six focus group interviews and five pair interviews between November 2019 and September 2020. For each group, three participants were recruited, but some groups received last‐minute cancellations for personal reasons. The participants were interviewed in groups containing the same type of professionals (students, mentors, educators) because students did not feel free to share their challenging experiences in the same group with mentors and/or educators. One researcher (VV) conducted all interviews. The themes of the interviews were based on previous research literature (FNAE, Finnish National Agency for Education., 2018; Immonen et al., 2019) and the research question. The interview topics were: (1) content of assessment during work‐based learning; (2) methods used to obtain information about the practical nursing students' competence; (3) environment for work‐based learning and competence assessment; (4) roles of the student, mentor and organization; (5) aspects that increase the success of mentoring and assessment; (6) aspects that decrease the success of mentoring and assessment. Because of the rich data, competence assessment and evaluation will be further described in a later publication. The interviews were recorded with the consent of the study participants and lasted 95–151 min.

### 2.4 Data analysis

The research data were analysed by inductive content analysis. This is a systematic and objective process which yields descriptions of the research phenomenon on a theoretical level (Kyngäs, 2020a). The analysis was assisted by Nvivo data management software (V.11; Alfasoft, Göteborg, Sweden). The recorded interviews were transcribed word‐for‐word into text format, after which the researchers carefully studied the material by listening to it once and reading through it twice. A sentence or statement was chosen as the unit of analysis. Next, all of the interview expressions that were related to the research question were retrieved and reduced without losing the original meaning. Data describing the students', mentors' and educators' experiences and perceptions yielded a total of 581, 479 and 581 codes, respectively. After that, the codes of all participants with similar content were combined into sub‐categories (*n* = 152), and these sub‐categories were further merged into categories (*n* = 37). At the final stage of analysis, the 37 categories were combined into five main categories (See Table 1). One researcher analysed the data by defining meaning units and codes and further continued in collaboration with other researchers defining sub‐categories, categories and main categories.

**TABLE 1:** Inductive content analysis.

Sub‐category	Category	Main category
Competence acquired at the vocational institution during theory teaching (E)	Student's previous competence	Student as a goal‐oriented and responsible learner
Competence acquired through previous work‐based learning (E)		
Work experience (S,M)		
Life experience (S,M)		
Respect (S)	Student's attitude towards learning	
Humility (S)		
Willingness to learn (S,M,E)		
Positive attitude towards mentoring (M,E)		
Flexibility (S)		
Behaviour (S,M,E)		
Interest in social‐ and health care work (S,M,E)	Student's motivation	
Interest in the workplace (S,E)		
Ensuring work safety (M)	Student's action as a learner	
Ensuring customer, resident and patient safety (S,M)		
Learning goal orientation (S,M,E)		
Compliance with rules and instructions (S,M,E)		
Activity (S,M,E)		
Taking responsibility for his−/her own learning (S,M,E)		
Working as a member of the work community (S,M,E)		
Communication with the educator (S,E)		
Competence in basic nursing skills (M)	Student's professional skills	
Ability to adapt and respond to changing work situations (M)		
Interpersonal skills (S,M)		
Language skills (S,M)		
Fairness (S)	Student's attributes and background	
Culture (S,M)		
Positivity (S)		
Age (E)		
Courage (S,M,E)		
Suitability for social‐ and health care work (E)		
Challenges in embracing new competence (M,E)	Student's learning difficulties	
Telling the mentor about learning difficulties (M,E)		
Receiving feedback (S,M,E)	Importance of feedback in the assessment of learning	
Giving feedback (M)		
Requesting feedback (S,M,E)		
Student's health (S)	Student's life situation	
Challenges of everyday life (M)		
Mentor's previous education (E)	Mentor's vocational competence	Mentor as a supporter and enabler of a student's goal‐oriented learning process
Work experience (S,E)		
Experience as a student mentor (M)		
Theoretical knowledge (S,M,E)		
Evidence‐based competence of care procedures (S)		
Interest in student mentoring (S,M,E)	Mentor's motivation	
Interest in nursing (S,M,E)		
Willingness to develop as a student mentor (M)		
Willingness to work in the workplace (E)		
Mentor's life situation (S)		
Optionality to act as a student mentor (M)		
Positivity (S)	Mentor's attributes	
Courageousness (S)		
Patience (S,M,E)		
Empathy (M,E)		
Approachable (S,M,E)		
Fairness (S,M,E)		
Familiar with the student's learning process (M)	Mentors support a student's learning process	
Individual student mentoring (S,M,E)		
Supportive of student mentoring (S,M,E)		
Integration of the theory and practice (S,M,E)		
Enabling learning situations (S,M,E)		
Creating a safe learning environment (S,M,E)		
Giving responsibility to the student (S,E)		
Consideration of patient safety in mentoring (S,M)	Considering safety in student mentoring	
Consideration of work safety in mentoring (S,M)		
Positive attitude towards student mentoring (S,M)	Mentor's interaction with the student and educator	
Positive attitude towards students (S)		
Collaboration with the educator (S,M,E)		
Functionality of the mentor‐student relationship (S)		
Goal orientation in mentoring (S,M,E)	Goal orientation in regular assessment of student's learning progress	
Giving constructive feedback to the student (S, E)		
Giving regular feedback to the student (S,M,E)		
Regular reflection with students during mentoring (M)		
Monitoring student learning progress (M)		
Mentor education (S,M,E)	Developing mentoring competence	
Asking the student for feedback on mentoring (M)		
Substantive knowledge (S)	Educator's competence	Educator as an instructor and supporter of a student's goal‐oriented learning process
Pedagogical skills (S)		
Inspiring (E)	Educator's attributes	
Positivity (E)		
Empathy (E)		
Friendly (E)		
Considerate (E)		
Fairness (S)		
Communication with the student (S,M,E)	Student mentoring	
Student support (S,E)		
Functionality of the student‐educator relationship (M)		
Availability to the student (S,M)		
Instructing the student in written assignments (S,E)		
Utilization of web platforms in student mentoring (S,E)		
Finding solutions to problem situations (S,M.E)	Educator's collaboration with the mentor and organization	
Mentor support (S,M,E)		
Collaboration with the mentor (S,M,E)		
Collaboration with the organization (M,E)		
Instructing the student in setting goals (S,E)	Ensuring a student's goal‐oriented learning	
Clarifying the vocational competence requirements to students (S,M,E)		
Clarifying the vocational competence requirements to mentors (S,E)		
Assessment of a student's learning progress (S,E)	Assessment of a student's learning progress	
Ensures that student receives feedback from the mentor (S,E)		
Increasing the mentor's mentoring skills (S,E)	Increasing the mentor's mentoring competence	
Increasing the mentor's assessment skills (E)		
Planning an individual study plan (S,E)	Takes into account a student's individuality	Vocational institution as an enabler of individual learning process
Directing the students to an appropriate workplace (S,M,E)		
Enabling optional study units for students in their studies (S)		
Taking into account students' individual wishes in the selection of workplace (S)		
Teaching care procedures at the vocational institution (S,M)	Preparing students for work‐based learning	
Theory teaching at the vocational institution (S,M)		
Psychological preparation of the students for work‐based learning (M)		
Ensuring students' suitability for social‐ and health care work (M)		
Ensuring student's readiness for work‐based learning (M,E)		
Clarifying vocational competence requirements and assessment criteria for students in vocational institution (S,M,E)		
Informing students about tasks, agreements and instructions related to the work‐based learning (M,E)		
Informing students about the assignments of the work‐based learning (S)		
Informing students about forms related to the work‐based learning (S)		
Collaboration with workplaces (M,E)	Collaboration with workplaces and other vocational institutions	
Collaboration with other vocational institutions (E)		
Concluding training and apprenticeship agreements for workplaces (S)		
Informing workplaces about changes in education (S)	Workplace information	
Informing workplaces about rules and instructions related to the work‐based learning (M,E)		
Ensuring that a student has been assigned a mentoring teacher for the work‐based learning (S)	Providing adequate resources	
Proper allocation of teacher resources (E)		
Educator's work planning allows for workplace visits (S,M,E)		
Ensuring reasonable amount of work‐based learning students per educator (E)		
Providing extra resources to the educator when needed (E)		
Organizing mentor education (S,M,E)	Developing student mentoring and assessment of learning	
Organizing feedback surveys for students and mentors (E)		
Drafting of written assignments related to the work‐based learning (E)		
Drafting of forms related to the work‐based learning (E)		
Ensuring patient safety (E)	Ensuring the quality of customer, resident and patient care	Workplace ensures secure learning environment
Taking care of the values of patient care (S)		
Taking care of employees' professional development and competence (S)		
Promoting evidence‐based nursing (S)		
Creating a positive atmosphere in the workplace has a significant impact on the student's enjoyment and learning (S,E)	Creating a positive workplace atmosphere	
Taking care of employee well‐being (S)		
Appointing each student one or two mentors (S,M,E)	Preparing for the student's work‐based learning period	
Informing the appointed student mentor about the student (M,E)		
Taking into account the student mentoring in staff's work planning (S,E)		
Involving the student in the work community (S)	Creating a secure learning environment	
Equal treatment of students (S)		
Welcoming the student kindly to the workplace (S,M)		
Creating a safe learning environment (M)		
The entire working community takes responsibility for student mentoring (S)		
Providing the mentor with enough time for student mentoring (S,M,E)	Providing adequate resources	
Providing the mentor with enough time for student's assessment (S,M,E)		
Providing the mentor with enough time for discussion with the student and the educator (E)		
Ensuring an adequate number of staff in the workplace (S)		
Enabling employees' rotation to act as a students' mentor (S,M)		
Management attaches importance to successful student mentoring (E)	Positive culture towards students and mentoring	
Immediate manager importance to successful student mentoring (S,E)		
Immediate manager knows how student mentoring in the workplace works (S)		
Development of student mentoring and assessment of learning (S,M,E)		
Collaboration with the vocational institution (E)	Collaboration with the vocational institution and educators	
Collaboration with the educators (E)		

*Note*: What kinds of experiences and perceptions do practical nursing students, mentors and educators have of student's successful work‐based learning and the assessment of learning progress? (S = student, M = mentor, E = educator).

### 2.5 Ethical considerations

The study was conducted in accordance with good scientific practice (TENK, 2019). Permission to conduct the study was obtained from each target organization. Before participating in the interviews, the students, mentors and educators received an e‐mail from their organization's contact person with information about the study. The letter contained information about the study purpose and aim, the interviews, the voluntary nature of participation, the anonymity of the subjects, and the confidentiality of the results. Written informed consent to participate in the study and to record the interviews was obtained from each participant. The interviews were conducted at a time that was convenient for all of the study participants. The researcher conducting the interviews (VV) ensured that she did not interview personal colleagues or previous students. The research material was stored as a password‐protected file in a database that only the researcher had access to; all of the material will be destroyed once it has served its purpose in the research project.

## 3 RESULTS

The five main categories that describe practical nursing students' successful work‐based learning and the assessment of learning progress are: (1) The student as a goal‐oriented and responsible learner. (2) The mentor as a supporter and enabler of a student's goal‐oriented learning process. (3) The educator as an instructor and supporter of a student's goal‐oriented learning process. (4) The vocational institution as an enabler of individual learning processes. (5) The workplace ensures a secure learning environment (see Table 1).

### 3.1 Student as a goal‐oriented and responsible learner

Student as a goal‐oriented and responsible learner included several distinct aspects, namely, the student's previous competence, attitude towards learning, motivation, action as a learner, professional skills, attributes and background, learning difficulties, feedback in the assessment of learning, and life situation.

The students and mentors reported that students' life experiences and possible previous work experience affect learning in the workplace and the assessment of learning. The educators mentioned that students have accumulated previous knowledge during their studies.

The participants (including students, mentors, and educators) expressed that a student's attitude was essential to successful work‐based learning. The mentors preferred to be responsible for students who have a positive mindset towards studying nursing and who are generally well‐behaved. Students valued student's humility, flexibility and respect for both patients and employees. The participants in this study articulated that a motivated student shows a genuine desire and interest to become a health and social care professional.

Skills that have been gained by a student who has been present at school and take care of their studies (Educator)

Then when a student is asked to pick up a towel, he/she sighs for a long time… (Mentor)

That the student seems to be interested in the nursing. It is really important that he/she shows that he/she is interested in caring for people. (Mentor)

The participants reported that a feeling of purpose in learning and the student taking action were important aspects of successful learning and the assessment of progress during work‐based learning. According to the interviews, a goal‐oriented, motivated student is active and takes responsibility for his/her own learning. During work‐based learning, the student must actively set learning goals which are based on vocational competence requirements. The student's professional skills were explained by students developing competence in basic nursing skills and the ability to adapt and respond to changing work situations. The students and mentors perceived that the quality of a student's interpersonal skills is directly related to the promotion of learning. The mentors viewed the student's poor language skills as a hindering factor in learning in the workplace. They were particularly concerned about the risk to patient safety because of the misunderstanding.

In the first week, I always tick what I already know. Which do not need so much to practice. Then I always check what is left. (Student)

The student should be able to speak to the elderly in their language. (Mentor)

According to the interview responses, a student's attributes and background involved their fairness, cultural background and positivity. Furthermore, the participants mentioned that a student's courage will affect his/her learning. A courageous student dares to encounter the work community and patients, and seek out different care situations. However, the student must listen to the mentor's instructions and act accordingly to ensure customer, resident and patient safety. Mentors and educators experienced that students' learning difficulties can be challenging for mentoring and teaching. They hoped that students would be open about their challenges in order to prevent misunderstandings. Additionally, the participants mentioned that one of the student's most important tasks is asking questions and receiving feedback. The interviewed participants shared that it was important for the student to comply with workplace rules and working hours.

A young student also needs the courage to do nursing. (Educator)

She was unable to absorb the knowledge on schedule. (Mentor)

The student can receive negative feedback from the mentor without being offended. (Student)

### 3.2 Mentor as a supporter and enabler of a student's goal‐oriented learning process

The second main category comprised a mentor's vocational competence, motivation, attributes, support of a student's learning process, considering safety in mentoring, along with student‐educator interactions, goal‐oriented in regular assessment of a student's learning progress, and developing their own mentoring competence. The participants who participated in the study considered the mentor's vocational competence to be an important aspect of successful learning. The mentors expressed that the competence and confidence to act as a mentor increase with work experience, while educators added that a mentor's theoretical knowledge was a significant part of their professional skills.

When I had no work experience, I was unsure if I could do it myself. (Mentor)

The participants perceived the mentor's motivation in student mentoring and assessment of learning as essential aspects of successful learning. The participants expressed that a mentors' attitude and motivation are influenced by their interest in nursing and the willingness to work. When discussing mentor attributes, the participants mentioned that a mentor should be patient and easy to approach. The mentors said that a competent mentor clearly states and justifies various issues to the student and gives the student time to learn. The participants also pointed out that a skilled mentor is fair towards the student.

The mentor wants to give the student knowledge and skills based on his/her own experience and view. (Educator)

A good mentor should also be patient. (Student)

The participants described that the mentor should be able to support each student's learning process, and to acknowledge the student's previous skills, the stage of their studies, and their individual way of learning. The participants mentioned enabling learning situations and integration of theory and practice as tasks for the mentor. These tasks contribute to students' learning in the workplace. The mentors and educators reported that students who were gradually given more responsibility would eventually be able to independently care for residents and patients. The mentors emphasized that customer and work safety is their responsibility, and mentoring must always consider patient safety. For this reason, the students commented that a mentor should closely monitor the work and provide assistance whenever necessary in the case that a student is provided more responsibility during work‐based learning.

When I have the student under my mentoring, I let him/her work as much as possible. (Mentor)

In addition to patient safety, student safety need also be considered. Because, there may be patients who are aggressive. Student safety is my responsibility. (Mentor)

The participants reported that the mentor should prepare for the student's arrival at the workplace and, in problem situations, be in contact with the educator. The mentor's interactions with the student and educator should also be visible in their daily mentoring practice. A functioning relationship requires for the mentor to have a positive attitude towards the students and mentoring. The educators experienced that the student's motivation to learn decreases if the student suspects that the mentor does not like them.

As a mentor, I could contact the educator more actively, for example by e‐mail. (Mentor)

The participants reported that it was important for the mentor to guide the student using goal‐oriented practices. As such, the students hoped that the mentor would discuss their goals with them on a regular basis and would give feedback constructive, objective and immediate. The educators and students emphasized the fact that a mentor should be aware of the vocational competence requirements and assessment criteria; this would translate into mentoring that is both goal‐oriented and in line with the assessment criteria. The participants reported that regular education is crucial to enhancing a mentor's mentoring skills and further development. The participants considered it important that organizations enable their employees to participate in mentoring education.

A person learns when he/she told if he/she has done something wrong. And even the positive feedback is nice. (Student)

Mentor education should be every six months. There will be a lot of new information. (Mentor)

### 3.3 Educator as an instructor and supporter of a student's goal‐oriented learning process

The third main category was explained by the educator's competence, the educator's attributes, student mentoring, the educator's collaboration with the mentor and organization, ensuring goal‐oriented learning among students, assessment of students' learning progress and increasing the mentor's mentoring competence. The students valued an educator's competence, i.e., substantive knowledge and pedagogical skills. A pedagogically skilled educator knows how to consider differences between students and their abilities to learn new things. An educator's attributes were commonly mentioned as an important aspect of successful learning. The students emphasized the importance of educators who were fair and treated all students in a similar way. The educators experienced that they should be considerate and emphatic in the workplace. They also mentioned that the educator can influence the workplace atmosphere by being positive, inspiring and friendly.

The educator speaks more slowly when the student's native language is not Finnish. (Student)

The educator can influence the atmosphere in the workplace by being friendly, inspiring, positivity. (Educator)

The participants mentioned that it was important for the educator to communicate, collaborate and visit the workplace twice during the student's work‐based learning. Both students and educators mentioned that the utilization of a web platform could benefit student mentoring. In the case that a problem arises, the participants articulated that the educator should contact the mentor either by calling or in person. The educators emphasized that the functionality of the mentor‐student relationship plays a significant role in the student's learning progress. The participants mentioned that an educator can ensure goal‐oriented learning among students by clarifying the vocational competence requirements and assessment criteria for students and mentors. Both the students and educators highlighted the fact that the educator is responsible for assessing practical nursing students.

It is important that the educator contacts the student for example by calling or WhatsApp. (Student)

It would be good for the educator to call a few times. For example, during five week work‐based learning, the educator could call after the first week and the again during the third week. (Mentor)

When the teacher visits the workplace, he/she should ensure that the student and the mentor understand the assessment criteria’. (Mentor)

During the visit the workplace, the educator asks the mentor how the student's learning has progressed. (Student)

The students and educators perceived that the educator is responsible for increasing the mentor's mentoring competence and assessment skills. This was particularly highlighted for situations in which the mentor has little experience in student mentoring or changes have been made to practical nursing education. The educators emphasized that they should encourage the mentor to participate in mentoring education.

It is the responsibility of the educator to clarify the mentor with the vocational competence requirements for the work‐based learning. So that the educator knows what he/she needs to teach the student and what he/she needs to assessment. (Educator)

### 3.4 Vocational institution as an enabler of individual learning process

The fourth main category consisted of six categories, namely, taking into account a student's individuality, preparing the student for work‐based learning, collaborating with workplaces and other vocational institutions, informing the workplace, providing adequate resources and developing student mentoring and the assessment of learning. The participants experienced that it was important to consider each student's individuality. As such, the student should be directed to a workplace that meets the vocational competence requirements. The educators emphasized that it was the responsibility of the vocational institution to ensure that each student's learning progressed according to an individual plan and that the student is ready for a period of work‐based learning.

Taking into account the student's previous competence when planning an individual study plan. (Student)

I hope the vocational institution will ensure the student is ready for the work‐based learning. (Mentor)

The students and mentors reported that preparing students for work‐based learning in a vocational institution involves informing them about tasks, agreements, instructions and forms related to work‐based learning. Vocational institutions often provide students with informational lectures before the work‐based learning period begins. The participants highlighted that one of the main tasks of a vocational institution is to clarify vocational competence requirements and assessment criteria to students.

The students and mentors also reported that the vocational institution is responsible for increasing students' theoretical knowledge. The students added that they should be taught concepts that are mentioned in the vocational competence requirements. The educators mentioned that the vocational institution is responsible for the psychological preparation of students for work‐based learning. Since demanding and unexpected situations can occur in the workplace, it is important for students to be aware of the potential reactions associated with these situations. The educators also reported that the vocational institution needs to ensure that each student is suitable for social‐ and health care work. The mentors and educators articulated that functional collaboration between the workplace and vocational institution is important. The students mentioned that the conclusion of training and apprenticeship agreements is the result of collaboration between the institution and workplaces. The educators commented that there could be more collaboration between vocational institutions in the development of work‐based learning and assessment of learning.

It is important that the collaboration between the vocational institution and the workplace is successful. (Educator)

The participants reported that the educator should have enough time to visit the workplace and adequate resources from the educational institution. The educators also emphasized that, if necessary, additional resources should be provided for visiting the workplace, for example, if there are challenges in mentoring a certain student. The educators mentioned that the mentoring and assessment shifts in the workplace occasionally cause challenges due to the educators' teaching obligations and the mentors' shift work. In addition, it is sometimes challenging for an educator to mentor many students at the same time. The mentors experienced problems with time management and regretted that they did not provide adequate and individual mentoring for students due to the poor allocation of resources. The participants reported that one of the tasks of the vocational institution is to organize education in order to develop student mentoring and assessment. The educators identified drafting written assignments and forms related to the work‐based learning as another task of the vocational institution. They also mentioned that the vocational institution should provide surveys to the students and mentors after work‐based learning to assess how the period met learning expectations.

The educator came to the workplace on her/him day off because she/he did not have time at other times. On Saturday. (Student)

Organizing mentor education more than once a year in the workplace so that as many employees as possible can take part in it. Although it is half a day. (Mentor)

### 3.5 Workplace ensures secure learning environment

The fifth category comprised ensuring the quality of customer, resident and patient care, creating a positive workplace atmosphere, preparing for the student's work‐based learning period, creating a secure learning environment, providing adequate resources, positive culture towards students and mentoring and collaboration with the vocational institution and educators. According to the students, the organization should take care of the values of patient care by ensuring the quality of customer, resident and patient care. A good organization takes care of the well‐being and professional development of its employees. The students experienced that they can best learn in an environment in which the nursing practices are based on up‐to‐date evidence. The educators mentioned that the organization should ensure patient safety across all activities, including student mentoring.

A good organization takes care of the well‐being of the employees, ergonomics, orientation and education of its employees and all… (Student)

The students and educators experienced that a good atmosphere is important for successful work‐based learning and its assessment. Students can expect to be treated positively in a workplace with a good atmosphere. Preparations for the students' work‐based learning period influence both students' experiences and learning outcomes. According to the participants, the organization should appoint each student with one to two mentors prior to the work‐based learning. The mentors highlighted the importance of creating a secure learning environment for students. According to both mentors and students, how the student is welcomed into a new environment has a significant effect on their learning process. As such, the student should be greeted with openness and kindness. The students also hoped that they would be treated equally in the workplace regardless of their background. They said that it is important to feel as though they are part of the work community.

The atmosphere in the workplace bad. And the student had to listen to the disagreements of the employees. An underage student was there to settle… (Educator)

It happened to me once as follows… When I came to work in the morning and sat the table, I was told: do you go with that student to measure patients' blood sugar? I was amazed at whom the student was? From whom is blood sugar measured? Is the student that boy there*?* (Mentor)

The workplace is bad if no one employees notices you when you start the work‐based there and you get to be alone. Nobody says anything. They just wondered if some student become to the workplace again. The student immediately gets the feeling that the workplace is not good. (Student)

The participants articulated that the organization should provide adequate resources for work‐based learning, i.e., enough time for student mentoring and the assessment of learning. The mentors shared that they do not always have sufficient time for mentoring because of a busy work schedule. In addition, they stated that they often do not have enough time to read the vocational competence requirements and assessment criteria. The students and mentors reported that occasional staff shortages in the workplace decrease the time for student mentoring and assessment. Both the students and mentors shared that mentors take turns mentoring students. This prevents the mentors from getting tired of mentoring. The students also pondered whether every mentor is able to inform their immediate manager in case they find the mentoring to be taxing.

I told the mentor that I would like to learn. There was a hurry. He/she said there is a little time. The mentor does the work in 15 min, but if she/he has to teach the work to the student, it takes more than 30 min. In a hurry, the student is not allowed to practice. (Student)

A positive culture towards students and mentoring had a strong association with a manager's role in mentoring. The educators experienced that the positive attitude of management towards students and student mentoring will greatly affect how the entire work community reacts towards, and treats, students. The participants reported that the organization should permit employees to participate in mentoring education. The participants greatly highlighted the collaboration between the institution and workplaces. The educators emphasized that there should be a low threshold for contacting them at the workplace. The educators mentioned the job title of clinic educator, which they felt was an excellent example of collaboration between an educational institution and an organization.

There are workplaces where the students and mentoring are important. Students are considered future colleagues. (Educator)

Then the student knows the name of his/her mentor and what phone number he/she will call. (Educator)

## 4 DISCUSSION

The aim of this study was to describe practical nursing students', mentors' and educators' perceptions of learning and assessment of it in the context of work‐based learning. The participating students appreciated completing their studies in a workplace with a good atmosphere because a positive atmosphere means that the students will also be treated positively. Papastavrou et al. (2016) stated that the inclusion of students in the work community increases student satisfaction with the clinical learning environment. The students participating in the present study also experienced that it was important for them to feel as though they were part of the work community. A student's good interpersonal skills and courage promote learning because the student will seek out different members of the work community and get involved in various care situations. Goh (2021) has researched student teachers' learning at school placements and university in a school‐university partnership. She mentions that the education of the student teachers required students to move simultaneously between the two learning sites, which either reinforced or changed their existing teacher identities. This same thing can be observed in the education of practical nurses.

Previous research has shown that a mentor's motivation, professionalism, and pedagogical skills contribute to successful mentoring (Karjalainen et al., 2015; Tuomikoski et al., 2018; Tuomikoski, Ruotsalainen, Mikkonen, & Kääriäinen, 2020). Participants in this study valued a mentor's professionalism. The participants expressed that mentors should guide students on an individual level in that they consider the student's way of learning, prior learning, work experience and specific life situation. Papastavrou et al. (2016) also found that individual mentoring is associated with student satisfaction. The study participants felt that it was important to provide students with regular and constructive feedback that is related to the vocational competence requirements. Regular discussion with students was also considered important for student learning. These findings agree with previously published results, as reflective discussions and receiving feedback have been linked to student learning and satisfaction (Cassidy et al., 2012; Pitkänen et al., 2018). The participants in this study mentioned that active and goal‐oriented students will request feedback from their mentors. A supportive mentor will gradually increase a student's responsibility to reflect on their work‐based learning; however, they will ensure patient and occupational safety at all times.

According to several studies, a mentor's training significantly increases their mentoring ability and assessment skills (Immonen et al., 2019; Ruuskanen et al., 2018; Tuomikoski et al., 2018; Tuomikoski et al. 2020b). The participants reported that the vocational institution is responsible for organizing mentoring education. The educators commented that they should encourage the mentors to participate in mentoring education. According to Arkan et al. (2018), students have expressed a desire for more practice in care procedures. Furthermore, the same students experienced that the exercises they completed during their studies were somewhat inconsistent with the activities of practical nursing staff. This was also observed among the students interviewed in the present study, who considered practising care procedures at the vocational institution to be an important part of their studies.

The participants considered the nomination of one or two mentors for each student to be an essential component of successful work‐based learning and assessment. Practical nursing students most often work in workplaces where schedules are organized using three shifts. The mentors reported that the appointment of two mentors per student would mean that one of the mentors could work with the student while the other is free. Sharing responsibility for mentoring and assessment also increases a mentors' ability to act as a student mentor. Papastavrou et al. (2016) have found that the nomination of a student mentor for each student increases student satisfaction.

In the present research, collaboration between the student, mentor, and educator was considered important to the progression of studies (Goh, 2022) and the assessment of learning. Several previous studies have also highlighted the student‐mentor relationship when discussing clinical learning (Immonen et al., 2019; Papastavrou et al., 2016). The participants in this study considered it important that the student and educator, and the mentor and educator, show a strong working relationship. Collaboration may be more successful if the educator is actively connected to the workplace, a dynamic that was also mentioned by Helminen (2017). The mentors who participated in this study experienced that they could be more active in their communication with educators.

Previous research (Butler et al., 2011; Cassidy et al., 2012) has revealed excessive workloads among mentors when considering clinical nursing care, professional responsibility, and student mentoring; these activities, which include assessing their students' competence, require a lot of time. Cassidy et al. (2012) mentioned that the continuous turnover of students and the fact that these students are at various points in their studies has increased mentors' workloads. This stressful amount of work was also observed in the present study, as mentors felt that there is sometimes so much pressure at work that they cannot let a student practice at their own pace. The rush arising from a large number of patients, increased care requirements, and high patient turnover has also weakened the work atmosphere (Arkan et al., 2018). The participants of this study also highlighted that educators must be provided with adequate resources. For example, the educator should have enough time to visit the workplace to mentor students and support the mentor.

It has previously been mentioned that the available assessment documents and criteria use unclear language (Butler et al., 2011; Helminen, 2017). In the study by Cassidy et al. (2012), mentors described the language used in assessment criteria as sprawling, verbose and very confusing. In this study, the students and educators emphasized the fact that a mentor should be aware of the vocational competence requirements and assessment criteria. This is relevant because each student's learning process is based on national vocational competence requirements.

### 4.1 Trustworthiness of the study

The findings were reported using the Standards for Reporting Qualitative Research (SRQR; O'Brien et al., 2014) to strengthen transparency and trustworthiness. The phenomenon was studied by including three different groups of participants, which strengthens the understanding of the topic. The data were collected from certain settings (i.e. social‐ and health care organizations) in which practical nurses participate in work‐based learning, but excluded other relevant settings, such as kindergartens and schools. In the future, it would be necessary to extend the research to all organizations in which practical nursing students complete work‐based learning.

## 5 CONCLUSIONS

Vocational competence requirements and assessment criteria guide student learning and assessment in work‐based placements. During work‐based learning, a student's progress should be assessed by giving them regular feedback that corresponds to the vocational requirements and student's goals. The interview material revealed that an educator's visits to the workplace are essential and welcome. Regular communication with the mentor ensures that an educator can follow each student's goal‐oriented learning process. It is also beneficial for the mentor to actively communicate with the educator; this is particularly important in instances where there are certain challenges to a student's learning.

The workplace is responsible for providing support to mentors, arranging enough time for mentoring, and promoting participation in mentoring education. The workplace should also take care of the value base of patient care, well‐being at work and the implementation of evidence‐based knowledge into practice. A positive environment and secure learning environment are crucial characteristics of an effective workplace.

The results of this study can be used to develop the mentoring and assessment of practical nursing students' progress in work‐based learning. As there is no previous scientific research on the mentoring and assessment of practical nursing students, more empirical evidence from different organizations and settings is urgently needed.

## AUTHOR CONTRIBUTIONS

Study design: Virpi Välimaa, Anna‐Maria Tuomikoski, Kristina Mikkonen. Data collection: Virpi Välimaa. Data analysis: Virpi Välimaa, Kristina Mikkonen. Manuscript writing: Virpi Välimaa, Anna‐Maria Tuomikoski, Kristina Mikkonen.

## FUNDING INFORMATION

The Finnish Association of Nursing Research (HTTS), 3800 é. Finnish Nurses Association (Suomen Sairaanhoitajat ry), 1000 é.

## CONFLICT OF INTERESTS STATEMENT

The authors have no conflict of interest to declare.

## ETHICAL APPROVAL

Research permission has been granted according to the Finnish data protection regulations. Ethical committee approval was not required.

## Data Availability

The data that support the findings of this study are available on request from the corresponding author. The data are not publicly available due to privacy or ethical restrictions.

## References

[nop21849-bib-0001] Arkan, B. , Ordin, Y. , & Yilmaz, D. (2018). Undergraduate nursing students' experience related to their clinical learning environment and factors affecting to their clinical learning process. Nurse Education in Practice, 29, 127–132. 10.1016/j.nepr.2017.12.005 29274524

[nop21849-bib-0002] Aussizz group . (2023). Diploma of Nursing in Australia. Study Diploma of Nursing Course in Australia|Aussizz Group. [last accessed 18.1.2023].

[nop21849-bib-0003] Billett, S. (2004). Workplace participatory practices: Conceptualising workplaces as learning environments. Journal of Workplace Learning, 16(6), 312–324. 10.1108/13665620410550295

[nop21849-bib-0004] Bowling, A. (2014). Research methods in health: Investigating health and health services (4th ed., p. 209). Open University Press.

[nop21849-bib-0005] Butler, M. P. , Cassidy, I. , Quillinan, B. , Fahy, A. , Bradshaw, C. , Tuohy, D. , O'Connor, M. , Mc Namara, M. C. , Egan, G. , & Tierney, C. (2011). Competency assessment methods – Tool and processes: A survey of nurse preceptors in Ireland. Nurse Education in Practice, 11(5), 298–303. 10.1016/j.nepr.2011.01.006 21324419

[nop21849-bib-0006] Cassidy, I. , Butler, M. B. , Quillinan, B. , Egan, G. , Mc Namara, M. C. , Tuohy, D. , Bradshaw, C. , Fahy, A. , Connor, M. O. , & Tierney, C. (2012). Preceptors´ views of assessing nursing students using a competency based approach. Nurse Education in Practice, 12(6), 346–351. 10.1016/j.nepr.2012.04.006 22640780

[nop21849-bib-0007] Dimitriadou, M. , Papastavrou, E. , Efstathiou, G. , & Theodorou, M. (2015). Baccalaureate nursing students' perceptions of learning and supervision in the clinical environment. Nursing & Health Sciences, 17(2), 236–242. 10.1111/nhs.12174 25377993

[nop21849-bib-0008] FNAE, Finnish National Agency for Education . (2018). Näytöt ja Osaamisen Arviointi (Competence Demonstrations and Assessment of Competence). https://eperusteet.opintopolku.fi/eperusteet‐service/api/dokumentit/4614532

[nop21849-bib-0009] FNAE, Finnish National Agency for Education (2017). Vocational Qualification in Social and Health Care https://eperusteet.opintopolku.fi/#/en/esitys/3689879/reformi/tiedot.

[nop21849-bib-0010] Goh, A. Y. S. (2014). Insights from a Bourdieusian lens: The relationship between college‐based and workplace learning in becoming a vocational‐technical education teacher in Brunei. Journal of Workplace Learning, 26(1), 22–38. 10.1108/JWL-06-2013-0034

[nop21849-bib-0011] Goh, A. Y. S. (2021). Learning cultures: Understanding learning in a school**‐**university partnership. Oxford Review of Education, 47(3), 285–300. 10.1080/03054985.2020.1825368

[nop21849-bib-0012] Goh, A. Y. S. (2022). Learning journey: Conceptualising “change over time” as a dimension of workplace learning. International Review of Education, 68(1), 81–100. 10.1007/s11159-022-09942-0 35573159 PMC9077343

[nop21849-bib-0013] Gymnasieguiden . (2021). Vård‐Och Omsorgsprogrammet (Nursing and Care Program). http://www.gymnasieguiden.se/gymnasieprogram/VO

[nop21849-bib-0014] Helminen, K. (2017). Dissertations in Health Sciences. In Nursing students´ final Assessement in clinical practice. Perceptions of teachers, students and mentors (Vol. 403). University of Eastern Finland https://erepo.uef.fi/bitstream/handle/123456789/17854/urn_isbn_978‐952‐61‐2420‐9.pdf?sequence=1

[nop21849-bib-0015] Helminen, K. , Coco, K. , Johnson, M. , Turunen, H. , & Tossavainen, K. (2016). Summative assessment of clinical practice of student nurses: A review of the literature. International Journal of Nursing Studies, 53, 308–319. 10.1016/j.ijnurstu.2015.09.014 26522265

[nop21849-bib-0016] Hyvärinen, N. , Palonen, M. , & Åstedt‐Kurki, P. (2019). Ohjattu harjoittelu opiskelijamoduulissa. Hoitajien kokemuksia hoitoalan opiskelijoiden ohjaamisesta ja oppimisesta (nursing training in the clinical education ward: Nurses' experiences in counselling nursing students and their learning). Hoitotiede, 31(1), 16–27.

[nop21849-bib-0017] Immonen, K. , Oikarainen, A. , Tomietto, M. , Kääriäinen, M. , Tuomikoski, A.‐M. , Kaučič, B. M. , Filej, B. , Riklikiene, O. , Vizcaya‐Moreno, M. F. , Pérez‐Cañaveras, R. M. , De Raeve, P. , & Mikkonen, K. (2019). Assessment of nursing students' competence in clinical practice: A systematic review of reviews. International Journal of Nursing Studies, 100, 1–13. 10.1016/j.ijnurstu.2019.103414 31655385

[nop21849-bib-0018] Kälkäjä, M. , Ruotsalainen, H. , Sivonen, P. , Tuomikoski, A.‐M. , Vehkaperä, A. , & Kääriäinen, M. (2016). Opiskelijaohjauskäytännöt, −resurssit ja ohjaajat terveysalalla: opiskelijaohjaajien näkökulma (Student counselling practices, resources and mentors in the health industry ‐ a student mentor's view). Hoitotiede, 28(3), 229–242.

[nop21849-bib-0019] Karjalainen, T. , Ruotsalainen, T. , Sivonen, P. , Tuomikoski, A.‐M. , Huhtala, S. , & Kääriäinen, M. (2015). Opiskelijaohjaajien arviot omasta opiskelijaohjausosaamisestaan (Mentors' assessment of their competence in mentoring). Hoitotiede, 27(3), 183–198.

[nop21849-bib-0020] King, R. , Ryan, T. , Wood, E. , Tod, A. , & Robertson, S. (2020). Motivations, experiences and aspirations of trainee nursing associates in England: A qualitative study. BMC Health Services Research, 20(802), 1–10. 10.1186/s12913-020-05676-7 PMC7448447 32847564

[nop21849-bib-0021] King, R. , Taylor, B. , Laker, S. , Wood, E. , Senek, M. , Tod, A. , Ryan, T. , Snowden, S. , & Robertson, S. (2022). A tale of two bridges: Factors influencing career choices of trainee nursing associates in England: A longitudinal qualitative study. Nursing Open, 9, 2486–2494. 10.1002/nop2.1266 35666045 PMC9374398

[nop21849-bib-0022] Komvuxutbildningar . (2021). Komvuxutbildning till Undersköterska . https://www.komvuxutbildningar.se/utbildning/komvuxutbildning‐underskoterska

[nop21849-bib-0023] Kyngäs, H. (2020a). Inductive content analysis. In H. Kyngäs , K. Mikkonen , & M. Kääriäinen (Eds.), The application of content analysis in nursing science research (pp. 13–21). Springer.

[nop21849-bib-0024] Kyngäs, H. (2020b). Qualitative research and content analysis. In H. Kyngäs , K. Mikkonen , & M. Kääriäinen (Eds.), The application of content analysis in nursing science research (pp. 3–11). Springer.

[nop21849-bib-0025] Malterud, K. , Siersma, V. D. , & Guassora, A. D. (2016). Sample size in qualitative interview studies: Guided by information power. Qualitative Health Research, 26(13), 1753–1760. 10.1177/1049732315617444 26613970

[nop21849-bib-0026] MEC, Ministry of Education and Culture, Finnish National Agency for Education . (2019). Finnish VET in a Nutshell. https://www.oph.fi/sites/default/files/documents/finnish‐vet‐in‐a‐nutshell.pdf

[nop21849-bib-0027] Nance, K. D. E. , & Brown, T. (2022). Use of curriculum mapping to promote licensed practical Nurses' attainment of a baccalaureate nursing degree. Journal of Nursing Education, 61(11), 646–649. 10.3928/01484834-20220912-07 36343195

[nop21849-bib-0028] NHS Healthcare assistant. https://www.healthcareers.nhs.uk/explore‐roles/healthcare‐support‐worker/roles‐healthcare‐support‐worker/healthcare‐assistant

[nop21849-bib-0029] O'Brien, B. C. , Harris, I. B. , Beckman, T. J. , Reed, D. A. , & Cook, D. A. (2014). Standards for reporting qualitative research: A synthesis of recommendations. Academic Medicine, 89(9), 1245–1251. 24979285 10.1097/ACM.0000000000000388

[nop21849-bib-0030] Papastavrou, E. , Dimitriadou, M. , Tsangari, H. , & Andreou, C. (2016). Nursing students' satisfaction of the clinical learning environment: A research study. BMC Nursing, 15(44), 1–10. 10.1186/s12912-016-0164-4 27436992 PMC4949757

[nop21849-bib-0031] Pitkänen, S. , Kääriäinen, M. , Oikarainen, A. , Tuomikoski, A. M. , Elo, S. , Ruotsalainen, H. , Saarikoski, M. , Kärsämänoja, T. , & Mikkonen, K. (2018). Healthcare students' evaluation of the clinical learning environment and supervision – A cross‐sectional study. Nurse Education Today, 62, 143–149. 10.1016/j.nedt.2018.01.005 29353088

[nop21849-bib-0032] Polit, D. F. , & Beck, C. T. (2021). Nursing research. Generating and assessing evidence for nursing practice. 11th Edition (Vol. 55, p. 802). Wolters Kluwer.

[nop21849-bib-0033] Ruuskanen, S. , Koota, E. , Timonen, L. , Haapa, T. , Lääperi, M. , Kääriäinen, M. , & Meretoja, R. (2018). Ohjaajakoulutusintervention vaikutus opiskelijaohjaajien itsearvioituun ohjausosaamiseen yliopistosairaalassa (Impact of educational intervention to mentors competence in mentoring students at the university hospital). Hoitotiede, 30(3), 191–202.

[nop21849-bib-0034] TENK . (2019). The ethical principles of research with human participants and ethical review in the human sciences in Finland. Finnish National Board on Research Integrity TENK Publications, 3, 7–23. https://tenk.fi/sites/default/files/202101/Ethical_review_in_human_sciences_2020.pdf

[nop21849-bib-0035] Tuomikoski, A.‐M. , Ruotsalainen, H. , Mikkonen, J. , & Kääriäinen, M. (2018). The competence of nurse mentors in mentoring students in clinical practice – A cross‐sectional study. Nurse Education Today, 71, 78–83. 10.1016/j.nedt.2018.09.008 30265858

[nop21849-bib-0036] Tuomikoski, A.‐M. , Ruotsalainen, H. , Mikkonen, J. , & Kääriäinen, M. (2020). Nurses' experiences of their competence at mentoring nursing students during clinical practice: A systematic review of qualitative studies. Nurse Education Today, 85, 1–15. 10.1016/j.nedt.2019.104258 31830638

[nop21849-bib-0037] Tuomikoski, A.‐M. , Ruotsalainen, H. , Mikkonen, K. , Miettunen, J. , Juvonen, S. , Sivonen, P. , & Kääriäinen, M. (2020). How mentoring education affects nurse mentors' competence in mentoring students during clinical practice – A quasi‐experimental study. Scandinavian Journal of Caring Sciences, 34(1), 230–238. 10.1111/scs.12728 31250935

[nop21849-bib-0038] Vizcaya‐Moreno, M. F. , Pérez‐Cañaveras, R. M. , Jiménez‐Ruiz, I. , & de Juan, J. (2018). Student nurse perceptions of supervision and clinical learning environment: A phenomenological research study. Enfermeria Global, 17(3), 319. 10.6018/eglobal.17.3.276101

